# A Phase I, Randomized, Single-Dose Study to Evaluate the Biosimilarity of HOT-3010 to Adalimumab Among Healthy Chinese Male Subjects

**DOI:** 10.3389/fphar.2021.646171

**Published:** 2021-03-17

**Authors:** Hong Zhang, Hong Chen, Xiaojiao Li, Min Wu, Xiaoxue Zhu, Cuiyun Li, Jingrui Liu, Haijing Wei, Yue Hu, Jingjing Wang, Yongmin Yang, Xiangyang Zhu, Yanhua Ding

**Affiliations:** ^1^ Phase I Clinical Research Center, The First Hospital of Jilin University, Jilin, China; ^2^ Shanghai Huaota Biopharmaceutical Co., Ltd., Shanghai, China

**Keywords:** adalimumab, biosimilar, immunogenicity, pharmacokinetics, intersubject variability

## Abstract

**Objective:** This study explored the bioequivalence of a proposed biosimilar HOT-3010 vs. its reference product (adalimumab) among healthy Chinese male subjects. The study also investigated the tolerance, immunogenicity, and pharmacokinetics (PK).

**Methods:** A randomized, double-blind, two-arm, parallel study was performed to examine the bioequivalence of HOT-3010 (40 mg) with that of adalimumab (Humira^®^, AbbVie) as a reference drug. The study subjects were followed up for 71 days.

**Results:** PK properties exhibited by HOT-3010 (*N* = 66) and adalimumab (*N* = 68) groups were similar. The 90% confidence intervals of the ratios for *C*_max_, AUC_0-*t*_, and AUC_0∞_ were observed to be in the range 80–125% on comparing the two groups. For anti-drug antibodies (ADA), the number of subjects found to be positive in the HOT-3010 group and adalimumab group were 29 (43.94%) and 32 (47.06%), whereas 27 (40.91%) and 27 (39.71%) subjects were found to be positive for NAb, respectively. Treatment-related treatment-emergent adverse events (TEAEs) were recorded in 32 subjects each in both the groups, respectively.

**Conclusion:** The PK characteristics and immunogenicity exhibited by HOT-3010 were similar to that of the reference product, adalimumab. The safety profiles were similar in both the treatment groups with mild-moderate adverse effects.

**Clinical Trial Registration:** http://www.chinadrugtrials.org.cn/index.html, identifier #CTR20181078.

## Introduction

Typically derived from living cells, biological products are different large and complex molecules. Owing to biologics’ molecular complexity and multifaceted production process, biosimilars are characterized differently from traditional small molecules (European Medicines Agency, 2015; US Food and Drug Administration, 2015; World Health Organization, 2015). Biologic therapies such as monoclonal antibodies are expensive and limit access worldwide (Zelenetz and Becker, 2016), despite significant therapeutic improvement. For about a decade, biosimilars are available in European and US markets, developed as copies of existing biologics.

A step-by-step approach for the development of biosimilars has been suggested by the US Food and Drug Administration (FDA), European Medicines Agency (EMA), and National Medical Products Administration (NMPA) (European Medicines Agency, 2015). The biological functional similarity was first assessed, followed by pharmacokinetic (PK) and pharmacodynamic (PD) properties, and at last, clinical similarity, including efficacy, safety, and immunogenicity was assessed, through the approved dose and pathway as that of the reference product (European Medicines Agency, 2015; US Food and Drug Administration, 2015; World Health Organization, 2015).

Adalimumab is specifically known to bind to tumor necrosis factor (TNF)-alpha and obstructs any interaction with the p55 and p75 cell surface TNF receptors. Surface TNF expressing cells are evidenced to be lyzed by adalimumab *in vitro* in the presence of complement. TNF, a naturally existing cytokine, participates in normal inflammatory and immune responses (Zelenetz and Becker, 2016; Fleischmann et al., 2019). The synovial fluid of rheumatoid arthritis (RA), juvenile idiopathic arthritis (JIA), psoriatic arthritis (PsA), and ankylosing spondylitis (AS) patients exhibit elevated levels of TNF, which participates in both the pathologic inflammation and joint destruction that are characteristic features of these diseases. High TNF levels also occur in psoriasis plaques. HUMIRA, a TNF blocker, is suggested for treating RA, JIA, PsA, and AS, and other diseases (AbbVieInc, 2020).

Worldwide, adalimumab biosimilars are being aggressively developed, such as MSB11022 and FKB327(Hyland et al., 2016; Puri et al., 2017). The reference product, as well as adalimumab biosimilar (HOT-3010), exhibit comparable primary structure, posttranslational modification, biochemical characteristics, and biological functions. Preclinical, PK and PD studies conducted on monkeys have revealed similarities (data not provided). Thus, the clinical development of HOT-3010 is imperative.

To establish their bioequivalence, assessing biological analogs and reference products through PK studies on humans is essential (China Food and Drug Administration, 2015). A single-dose PK investigation was conducted among healthy Chinese male volunteers for estimating the bioequivalence of HOT-3010 and adalimumab as control. Confounding factors such as disease conditions, comorbidities, and variability associated with treatment are prevented among healthy subjects. Although the reference drug’s therapeutic dose is 10–160 mg (AbbVieInc, 2020), the dosage for this study was set at 40 mg as per the sponsor's early clinical trial plan. The PK profiles of HOT-3010 and adalimumab (Humira^®^, AbbVie) were evaluated and compared while also assessing tolerability, safety, and immunogenicity of HOT-3010.

## Methods

### Study Design and Subjects

This investigation was performed in the phase I Clinical Research Center of the First Hospital of Jilin University between Nov 22nd, 2018, and June 07th, 2019 (Chinese Clinical Trial Registry, Registration No. CTR20181078). The Institutional Review Board of The First Hospital of Jilin University reviewed and approved all the related documents, such as the final protocol, any amendments, and informed consent. The declaration of Helsinki, International Conference on Harmonization Good Clinical Practice Guidelines, and local regulatory requirements were adhered to during this study. Written informed consent was obtained from the subjects before being enrolled in this study.

This randomized, double-blind, single-dose, two-arm, parallel study was conducted to explore the bioequivalence of the proposed biosimilar, HOT-3010, with its reference product (adalimumab) among healthy Chinese male subjects. The tolerance, immunogenicity, and pharmacokinetics (PK) of the reference product and the biosimilar were investigated. Overall, 136 eligible subjects were included and randomized at a ratio of 1:1 to be administered a single subcutaneous injection of 40 mg HOT-3010 or adalimumab. The sample was stratified by 70 kg weight. Subjects in each pre-assigned weight interval were equally allocated to the two treatment groups randomly.

For safety evaluation, all subjects were retained in the study center for at least 72 h following the dose administration. Thereafter, subjects were expected to return to the center at a scheduled time in the protocol and were followed up for 71 days.

Following were the inclusion criteria: 1) Healthy males in the age range 18–55 years; 2) with a body mass index (BMI) of 18.0–28.0 kg/m^2^; 3) total body weight in the range 55–90 kg; 4) and normal test reports or with no clinical significance for routine blood and urine and hepatic and renal functions examinations at the time of enrollment and absolute neutrophil count ≥1.8 × 10^9^/L.

The exclusion criteria comprised the following: 1) Subjects suffering from clinically significant diseases or with a history of the same; 2) positive for T-SPOT^®^ TB interferon-γ-release assays; were in contact with tuberculosis patients or/and presented suspected symptoms or signs of tuberculosis recently; 3) with a history of demyelinating diseases (including myelitis) 4) opportunistic infections (e.g., shingles); during the 6 months before screening; 5) systemic or local infection; 6) with serum triglyceride ≥3.42 mmol/L; and 7) antinuclear antibody titer ≥1:100.

The subjects were administered a single dose of subcutaneous injection of adalimumab 40 mg. They were randomized into two groups in a 1:1 ratio: HOT-3010 (Shanghai Huaaotai Biopharmaceutical Co. Ltd.; Batch number: 20181002) and adalimumab (Humira^®^, AbbVie Inc.), sourced from Europe. Batch number: 80318XH01) groups.

The subjects’ visit to the Clinical Research Unit for screening was scheduled 14 – 2 days before the dose was administered. They were admitted a day before the administration of the adalimumab biosimilar. The subjects observed fasting for at least 10 h before the biosimilar administration, who were then randomly allocated to two groups: the test (HOT-3010) and reference drug (adalimumab) groups.

### Pharmacokinetics Evaluations

At about an hour before administering the dose (pre-dose), blood samples were collected for PK evaluation, and at different time points up to 1,680 h (day 71) following dosing. The serum concentration of the adalimumab was evaluated by way of the Enzyme-linked immunosorbent assay (ELISA) method, and the assay was conducted at the Shanghai Xihua Testing Technology Service Co., Ltd. (Shanghai, China).

The PK parameters were calculated by utilizing a non-compartmental analysis model. The maximum observable serum concentration (*C*_max_), clearance (CL), half-life (t_1/2_), the volume of distribution (Vz), and area under the curve (AUC) from zero to the final quantifiable concentration (AUC_0−*t*_) and to infinity (AUC_0−∞_) values formed the concentration-time data. The PK analysis was performed by using actual sampling times. An internally validated software system, Phoenix WinNonLin^®^ v8.0 (Pharsight Corporation, Certara, L.P., Princeton, NJ, USA), was utilized for calculating PK parameters.

### Immunogenicity Evaluations

The anti-drug antibodies (ADAs) and neutralizing antibodies (NAb) were identified by examining blood samples collected at 1 h pre-dose and 15, 29, 43, and 71 days following the dose. ADA samples were evaluated by way of ELISA. The presence or absence of neutralizing antibodies was further assessed in ADA-positive samples using ELISA.

### Safety Evaluations

A combination of tests comprising physical examination, vital signs, electrocardiogram, and common laboratory tests such as urine analysis and chemistry was conducted for monitoring adverse events (AEs) and were documented and graded as per the National Cancer Institute Common Terminology Criteria for Adverse Events (V.4.03). Until the subjects with AEs normalized and attained a stable state, they were monitored (evaluated by the investigator and sponsor) or were lost to follow-up.

### Estimation of Sample Size

As per the current FDA guidelines, the geometric mean ratio (GMR) was set to be 95% to achieve 80% power (1−β) at the significance level (two-sided α = 5%). The coefficient of variation (CV) signifies intersubject variability (inter-CV). The sample size (initial: 108, inter-CV for adalimumab: 36%) was estimated with the help of NQuery 8.3.0.0 software (Boston, MA, United States) (US Food and Drug Administration, 2015), allowing for the 20% drop-out rate; the final sample group size was 136.

### Statistical Analysis

If the 90% confidence intervals were within the range 80–125% for *C*_max_, AUC_0−*t*_, and AUC_0−*∞,*_ bioequivalence was concluded. PK analysis set was utilized for conducting PK analysis for the study population. To adjust its effect on bioequivalence, weight was included in the analysis of variance (ANOVA) model as a fixed effect. Subjects who were administered the study drug were considered for safety analysis. Descriptive statistics for both PK parameters and demographical data were determined. *t*-test for normally distributed data and Wilcoxon rank test for data with unknown distribution was used for data analysis. SAS 9.4 Statistical Package (SAS Institute Inc., Cary, NC, United States) was utilized for conducting all statistical tests.

## Results

### Subjects

Two of the 136 subjects enrolled in the HOT-3010 group pulled out of the study owing to increased blood pressure ahead of the dose administration. Thus, only 134 subjects were administered the drugs and were considered for the safety and PK analyses sets, and 66 and 68 subjects were allocated to the HOT-3010 and adalimumab groups, respectively. A comparison of demographic and baseline characteristics between various treatment groups was performed, which were found comparable (Table 1).

**TABLE 1:** Demographics and baseline characteristics.

	HOT-3010 group (*n* = 66)	Adalimumab group (*n* = 68)	Total (*n* = 134)
Age (year), mean (SD)	37.7 (8.03)	38.2 (8.42)	38.0 (8.20)
Ethnicity (Han, n (%))	63 (95.4)	67 (98.5)	130 (97.0)
Weight (kg), mean (SD)	68.46 (8.859)	67.93 (6.634)	68.19 (7.785)
BMI (kg/m^2^), mean (SD)	23.5 (2.53)	23.9 (2.34)	23.7 (2.43)

### Pharmacokinetics Evaluations

**FIGURE 1:**
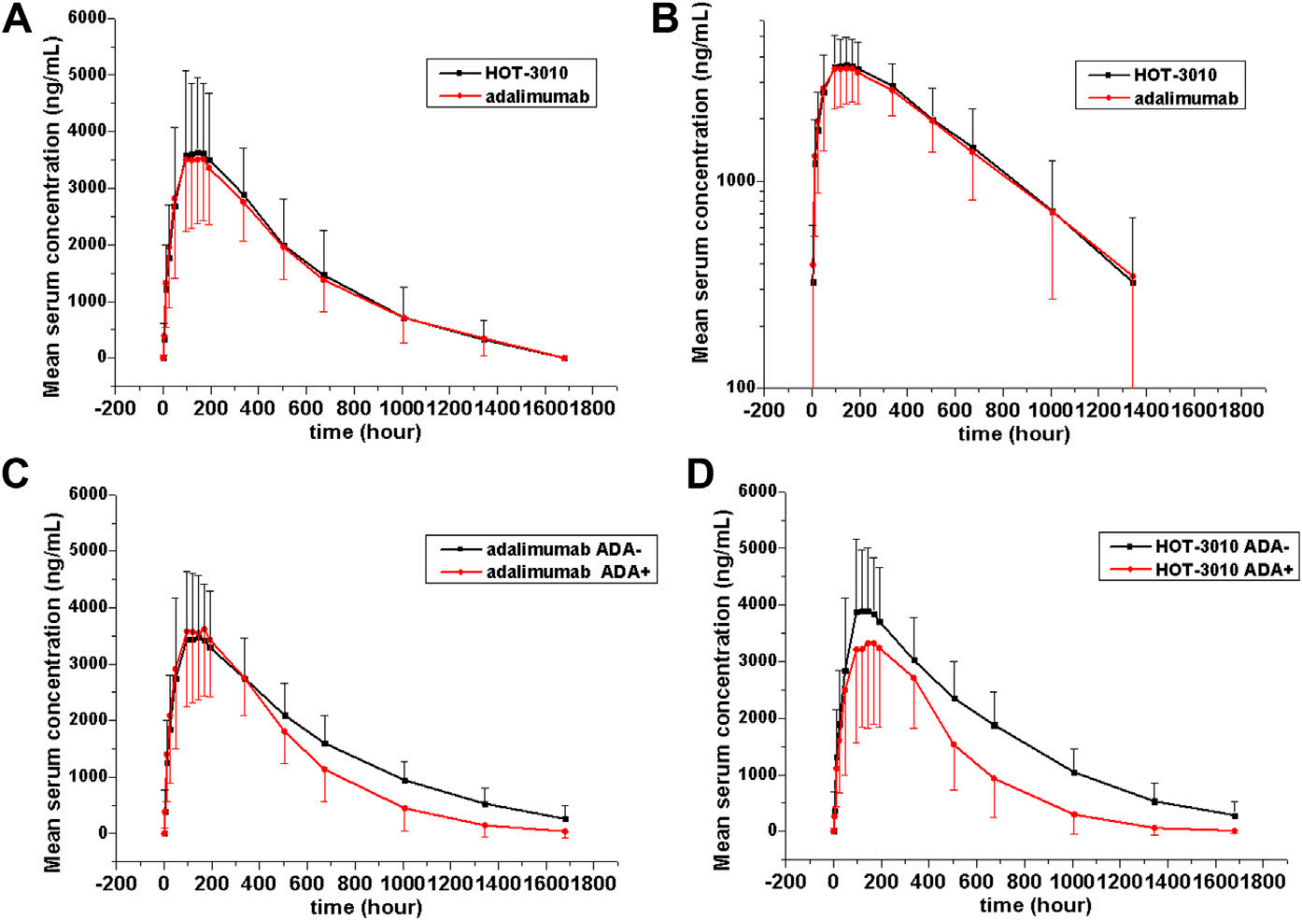
Serum drug concentration-time profile of adalimumab and its biosimilar HOT-3010 b y treatment following a single subcutaneous injection of 40 mg adalimumab and HOT-3010. Data are presented as Mean values ± standard deviation **(A)**; log10 mean values **(B)**; Mean values of ADA-positive and -negative individuals from the HOT-3010 group **(C)**; Mean values of ADA-positive and -negative individuals from the adalimumab group **(D)**.

Single-phase descent characteristics were exhibited by the mean serum concentration-time curve for the adalimumab and its biosimilar (Figure 1). The non-compartmental analysis revealed the slow clearance and longer *t*_1/2_ of adalimumab and its biosimilar. The median *T*_max_ values of the two groups at 144 h after the administration of the subcutaneous injection were found to be similar. The projected values of adalimumab mean *t*_1/2_ were also observed to be similar for the two groups, ranging from 285.94 to 308.23 h. The trends for CL and Vz values across the two groups were observed to be the same. The mean values of concentration-time profiles and *C*_max_ and estimates of AUC_0−*t*_ and AUC_0−∞_ and inter-CVs were found comparable, with their coefficient of variation values in the range 29.77–39.79% (*p* > 0.05, Table 2 and Figure 1).

**TABLE 2:** Pharmacokinetic parameters of adalimumab in each group (Mean [CV%] or median [min, max]).

	HOT-3010 group (*N* = 66)	Adalimumab group (*N* = 68)	*p*	GMR (90%CI)	GMR (90%CI)^a^	Re-estimated size
*T*_max_*(h)	144.00 (96.00, 336.02)	144.00 (48.00, 336.83)	0.45	—	—	—
*C*_max_ (ng/ml)	3904.35 (34.78)	3790.65 (33.55)	0.61	102.22 (92.06–113.49)	101.91 (91.72–113.23)	84
AUC0-t (hr*ng/mL)	2335328.82 (39.58)	2272030.28 (29.77)	0.65	98.47 (87.60–110.68)	98.82 (87.85–111.17)	104
AUC0-∞(hr*ng/mL)	2512012.43 (39.79)	2461783.86 (31.15)	0.74	98.27 (87.70–110.11)	98.65 (87.98–110.62)	106
t1/2(h)	285.94 (50.49)	308.23 (47.56)	0.38	—	—	—
CL/F (mL/h)	19.23 (50.26)	18.48 (44.87)	0.63	—	—	—
Vz/F (mL)	6847.75 (41.71)	7208.08 (33.13)	0.43	—	—	—

^a^ HOT-3010/adalimumab after excluding Subject no.004, whose ADA was positive pre-dose.

Pharmacokinetic comparisons for HOT-3010 vs. adalimumab have been presented. The ratio of geometric least-squares means for the HOT-3010 vs. adalimumab was 102.22, 98.47, and 98.27 for *C*_max_, AUC0-t, and AUC0-∞ and the 90% CI was 87.60–113.49 (Table 2). For all comparisons, 90% CI values of the *C*_max_, AUC0−t, and AUC0-_∞_ estimates were contained within the predefined 80.00–125.00% bioequivalence limits. Based on the outcomes of bioequivalence analysis (GMR and inter-CV), the sample size was reestimated and was observed to be less than the enrollment size, while only about 106 subjects met the bioequivalence analysis requirement (Table 2).

### Immunogenicity Evaluations

The existing ADA and NAb positive rates were observed to amplify with time, and the total positive rates of HOT-3010 and adalimumab for ADA (43.94 vs. 47.06%) and NAb (40.91 vs. 39.71%) were recorded to be the same at day 71. Precise results are presented in Table 3. No differences in the immunogenicity rates between the two groups were found. Subject no. 004 was recorded to be positive for ADA at pre-dose. Therefore, sensitivity analyses were conducted on excluding subject no. 004 to evaluate the influence of immunogenicity on the PK of the treatment, and the geometric mean values of *C*_max_, AUC_0−*t*_, and AUC_0−∞_ were similar among treatments. The serum concentration-time curves of HOT-3010 from 96 to 1,680 h and adalimumab from 504 to 1,680 h in ADA-positive subjects were lower than those in ADA-negative subjects (Figures 1C,D). The exposures were also lower for the ADA-positive subgroup than the ADA-negative subgroup in HOT-3010 and adalimumab group, except the *C*_max_ of adalimumab, due to the *C*_max_ values were similar between the ADA-positive and negative subgroup. However, the 90% CIs for the comparisons of *C*_max_, AUC_0−t_, and AUC_0−∞_ values were within the predefined bioequivalence limits of 80.00–125.00% regardless of ADA status (Table 2).

**TABLE 3:** ADA and Nab positive rates *n* (%) after subcutaneous injection of HOT-3010 or adalimumab.

Time (day)	HOT-3010 group (*n* = 66)		Adalimumab group (*n* = 68)	
	ADA positive rate	Nab positive rate	ADA positive rate	Nab positive rate
Pre-dose	1 (1.52)	0 (0)	0 (0)	0 (0)
15	0 (0)	0 (0)	6 (8.82)	0 (0)
29	9 (13.64)	3 (4.55)	11 (16.18)	2 (2.94)
43	12 (18.18)	10 (15.15)	7 (10.29)	6 (8.82)
71	28 (42.42)	27 (40.91)	28 (41.18)	27 (39.71)

ADA = anti-drug antibody; NAb = neutralizing antibody.

### Safety Evaluations

Of the total, 64 (47.8%) subjects who experienced treatment-related treatment-emergent adverse events (TEAEs), 32 (48.5%) subjects belonged to the HOT-3010, and 32 (47.1%) to the adalimumab group (Table 4). The overall incidence of treatment-related TEAEs was similar between the two groups. High blood triglycerides (6.1 vs. 4.4%), hyperuricemia (0 vs. 8.8%), total bilirubin increased (7.6 vs. 2.9%), alanine aminotransferase (3.0 vs. 5.9%), and aspartate aminotransferase (3.0 vs. 4.4%) and lowered neutrophil count (6.1 vs. 10.3%) were the common treatment-related TEAEs in the HOT-3010 as compared with adalimumab group, respectively.

**TABLE 4:** Treatment-emergent adverse drug reaction (number of events, the number (%) of subjects, more than 3%).

	HOT-3010 group (*n* = 66)		Adalimumab group (*n* = 68)	
	(*n*AE)	*n* (%)	(*n*AE)	*n* (%)
Total	53	32 (48.5%)	67	32 (47.1%)
Hypertriglyceridemia	5	4 (6.1%)	4	3 (4.4%)
Hyperuricemia	0	0	7	6 (8.8%)
Urinary tract infection	0	0	3	3 (4.4%)
Total bilirubin increased	7	5 (7.6%)	3	2 (2.9%)
Neutrophil counts decreased	5	4 (6.1%)	12	7 (10.3%)
Total bile acid increased	4	4 (6.1%)	1	1 (1.5%)
White blood cell count increased	2	2 (3.0%)	1	1 (1.5%)
Alanine aminotransferase increased	2	2 (3.0%)	6	4 (5.9%)
Urine protein-positive	2	2 (3.0%)	0	0
Aspartate aminotransferase increased	2	2 (3.0%)	4	3 (4.4%)
Neutrophil count increased	2	2 (3.0%)	1	1 (1.5%)
Headache	2	2 (3.0%)	0	0
Oropharyngeal pain	2	2 (3.0%)	3	3 (4.4%)
Cough	0	0	3	3 (4.4%)

The treatment-related Grade-III TEAEs occurred as a case of tuberculosis and infectious pneumonia in the HOT-3010 group (SAE) and a case of increased lipase and diarrhea in the adalimumab group. On day 71, subject no. 015 from the HOT-3010 group was diagnosed with tuberculosis, which was identified to study drug-related. Adalimumab likely suppressed the immune function, consistent with the label, which may have caused the body to be susceptible to tuberculosis. The treatment-related Grade-IV TEAEs emerged as a single case of hyperuricemia in the adalimumab group. The other treatment-related TEAEs were Grade I–II. All these AE cases were recovered by the last visit, which mostly did not require drug therapy, while no TEAE occurred that caused withdrawal.

The two drugs were found to be safe, and no remarkable differences between the two groups were observed. ADA development was not found to be correlated to TEAEs. None of the subjects exhibited clinically significant or serious hypersensitivity or anaphylaxis or injection site reaction following dosing. The Institutional Review Board of The First Hospital of Jilin University recorded all the drug-induced AEs.

## Discussion

Through this single-dose, phase I study of HOT-3010 administered in the form of 40 mg subcutaneous adalimumab injection, bioequivalence was established. The *C*_max_ and AUC values between the two treatment groups were compared; ANOVA results exhibited that the 90% CIs of the geometric means ratios for the PK parameters in the range 87.60–113.49%, and were all observed to be within the predefined bioequivalence interval 80–125% for the natural log-transformed data for each comparison. Other PK *T*_max_ and *t*_1/2_ parameters were also similar between the two treatment groups. HOT-3010 and adalimumab exhibited similar safety and immunogenicity profiles; all treatment-related TEAEs were observed to be mild to moderate in severity, with the absence of local reactions, evidencing that the healthy subjects well tolerated the two drugs. The outcomes of this investigation supported the utilization of the biosimilars in the subsequent clinical studies (Hyland et al., 2016; Puri et al., 2017; Hillson et al., 2018; von Richter et al., 2019).

Adalimumab was absorbed and distributed slowly. The *C*_max_, *T*_max_, and AUC values were reported as 4.7 ± 1.6 μg/ml and 131 ± 56 h, and 2167.38 ± 975 h*μg/mL, respectively, and the mean t_½_ equals ∼2 weeks following administration a single-dose of adalimumab 40 mg subcutaneously to healthy adult Caucasian subjects (Hyland et al., 2016; AbbVieInc, 2020). The PK properties of adalimumab were observed to be linear over the dose range 0.5–10.0 mg/kg following a single IV dose (Hyland et al., 2016; AbbVieInc, 2020). The PK parameters such as absorption, distribution, and adalimumab metabolism among the healthy study population were comparable with those in the literature data (Table 2).

PK analyzes in RA patients displayed an evident trend toward higher clearance of adalimumab in the presence of anti-adalimumab antibodies and poorer clearance with an increase in age among patients in the age range ≥40 years (Hyland et al., 2016; Puri et al., 2017; Hillson et al., 2018; von Richter et al., 2019). ADA certainly influenced drug concentration, and the ADA-positive group exhibited lower concentration within a certain time frame and lower exposure of adalimumab and its biosimilar, such as AUC, although it did not affect the bioequivalence results of this study (Table 2). This is similar to previous research reported by Elizabeth Hyland (Hyland et al., 2016). This appears to be related to higher clearance and the design of the PK assay, due to which the measurement of adalimumab becomes difficult when bound to ADA with neutralizing capability (Hyland et al., 2016). ADA is identified in approximately 10–25% of patients administered adalimumab without methotrexate (Poddubnyy and Rudwaleit, 2011). Almost 40% of the healthy treated subjects in this investigation formed a detectable ADA and NAb, with comparable outcomes for each treatment group. Considering the actual differences between healthy subjects and patients, further investigations are imperative, while the variations between this and earlier studies may be attributed to the high sensitivity of the investigative techniques utilized in the current one.

Four subjects from the HOT-3010 and six from the adalimumab group underwent at least one combined drug therapy for TEAEs during this research. The drugs used were analgesic tablets, amkahuang min capsule, loratadine, clindamycin, and so on. Adalimumab is a biological product with no influence by liver enzymes on its metabolic pathway. Thus the combination of these drugs was considered less likely to influence the outcomes of PK analysis of adalimumab (AbbVieInc, 2020). The inter-CV of adalimumab was found to be approximately 30–39% (medium), with a realistic sample size in this study, while about 53 subjects were proposed to be included in future bioequivalence studies (Hyland et al., 2016; Wynne et al., 2016; Kaur et al., 2017; Park et al., 2017; Puri et al., 2017; Shin et al., 2017; Hillson et al., 2018; von Richter et al., 2019; Cao et al., 2020).

Sixty-four (47.8%) subjects experienced treatment-related TEAEs and were mainly the mild (grade I-II) abnormality of the laboratory test value, such as reduced neutrophil count. Noteworthy are the incidents of treatment-related TEAEs were comparable between the HOT-3010 and adalimumab groups (48.5 vs. 47.1%, respectively), and nearly all of them recovered spontaneously by the last visit to the institute. Adeep Puri (Puri et al., 2017) found that 97 subjects (53.9%) experienced AEs that were related to adalimumab in an FKB327 biosimilar study in healthy subjects; the incidence was slightly higher than that in our study. The AEs (incidence of at least 10%) most often reported in the label after long-term treatment comprised infections (e.g., upper respiratory, sinusitis), injection site reactions, headache, and rash (Scheinfeld, 2005). The treatment-related TEAEs of this study were mild abnormalities of the laboratory indicator, rather than infection-related adverse reactions caused by immune suppression after long-term treatment. Although the incidence is higher, they were mild in this study after one dosage, similar to the literature (Puri et al., 2017).

Adalimumab is also known to modulate biological responses induced or regulated by TNF, such as modifications in the adhesion molecules’ levels responsible for leukocyte migration (AbbVieInc, 2020). Adalimumab perhaps influenced the distribution between blood and tissues such as neutrophils and white blood cells. Reduction in neutrophil count occurred in about 6.1–10.3% of subjects in this study, which rapidly normalized as drug concentrations dropped. As anti-TNF therapy is linked to immune suppression, AEs such as opportunistic infections are of special interest for this drug class (Zhang et al., 2017). Only one subject suffered from tuberculosis and infectious pneumonia. Although chest X-ray and T-SPOT^®^. TB interferon-γ-release assays resulted in negative at screening; an occult infection of tuberculosis was suspected by the investigators to recur when the body’s immune function was found to be reduced. Perhaps this subject was in touch with a tuberculosis patient during the treatment when his immunity was low and was thus infected (Zhang et al., 2017).

Overall, safety and tolerability of HOT-3010 and reference Humira^®^ (AbbVie) were reported in this study; all AEs were mild to moderate with no severe AEs observed, indicating good tolerance of these products (Fleischmann et al., 2019; Zhang et al., 2020).

## Conclusion

PK profiles of adalimumab biosimilars (HOT-3010) and adalimumab were thus demonstrated to be similar. The adalimumab biosimilars had almost comparable ADA and NAb profiles and a similar safety profile to that of the reference drug. The inter-CV of adalimumab was observed to be moderate among Chinese subjects. The clinical development of HOT-3010 as adalimumab biosimilars is well-supported by these data.

## Data Availability

The original contributions presented in the study are included in the article, further inquiries can be directed to the corresponding author.

## References

[B1] AbbVieInc (2020). Humira (adalimumab) injection for subcutaneous use. North Chicago, IL: AbbVie Inc Available at: https://www.accessdata.fda.gov/drugsatfda_docs/label/2020/125057s418s419lbl.pdf (Accessed December 16, 2020).

[B2] Cao G. Yu J. Wu J. Wang J. Xue Y. Yang X. (2020). A randomized, double‐blind, parallel‐group, phase 1 clinical trial comparing the pharmacokinetic, safety, and immunogenicity of the biosimilar HS016 and the originator adalimumab in Chinese healthy male subjects. Clin. Pharmacol. Drug Dev. 10 (3), 317–325. 10.1002/cpdd.816 32463599 PMC7984335

[B3] China Food and Drug Administration (2015). Scientific considerations in demonstrating bioequivalence to a reference product. Available at: http://www.sda.gov.cn/WS01/CL1751/147583.html (Accessed February 28, 2015).

[B4] European Medicines Agency (2015). Guideline on similar biological medicinal products. Available at: http://www.ema.europa.eu/docs/en_GB/document_library/Scientific_guideline/2014/10/WC500176768.pdf (Accessed August 25, 2015).

[B5] Fleischmann R. M. Genovese M. C. Enejosa J. V. Mysler E. Bessette L. Peterfy C. (2019b). Safety and effectiveness of upadacitinib or adalimumab plus methotrexate in patients with rheumatoid arthritis over 48 weeks with switch to alternate therapy in patients with insufficient response. Ann. Rheum. Dis. 78, 1454–1462. 10.1136/annrheumdis-2019-215764 31362993 PMC6837258

[B6] Fleischmann R. Pangan A. L. Song I. H. Mysler E. Bessette L. Peterfy C. (2019a). Upadacitinib versus placebo or adalimumab in patients with rheumatoid arthritis and an inadequate response to methotrexate: results of a phase III , double‐blind, randomized controlled trial. Arthritis Rheumatol. 71, 1788–1800. 10.1002/art.41032 31287230

[B7] Hillson J. Mant T. Rosano M. Huntenburg C. Alai-Safar M. Darne S. (2018). Pharmacokinetic equivalence, comparable safety, and immunogenicity of an adalimumab biosimilar product (M923) to Humira in healthy subjects. Pharmacol. Res. Perspect. 6. 10.1002/prp2.380 PMC5817835 29417761

[B8] Hyland E. Mant T. Vlachos P. Attkins N. Ullmann M. Roy S. (2016). Comparison of the pharmacokinetics, safety, and immunogenicity of MSB11022, a biosimilar of adalimumab, with Humira(®) in healthy subjects. Br. J. Clin. Pharmacol. 82, 983–993. 10.1111/bcp.13039 27285856 PMC5137823

[B9] Kaur P. Chow V. Zhang N. Moxness M. Kaliyaperumal A. Markus R. (2017). A randomised, single-blind, single-dose, three-arm, parallel-group study in healthy subjects to demonstrate pharmacokinetic equivalence of ABP 501 and adalimumab. Ann. Rheum. Dis. 76, 526–533. 10.1136/annrheumdis-2015-208914 27466231 PMC5445997

[B10] Park K. R. Chung H. Yang S. M. Lee S. Yoon S. H. Cho J. Y. (2017). A randomized, double-blind, single-dose, two-arm, parallel study comparing pharmacokinetics, immunogenicity and tolerability of branded adalimumab and its biosimilar LBAL in healthy male volunteers. Expert Opin. Investig. Drugs 26, 619–624. 10.1080/13543784.2017.1307339 28290731

[B11] Poddubnyy D. Rudwaleit M. (2011). Efficacy and safety of adalimumab treatment in patients with rheumatoid arthritis, ankylosing spondylitis and psoriatic arthritis. Expert Opin. Drug Saf. 10, 655–673. 10.1517/14740338.2011.581661 21554150

[B12] Puri A. Niewiarowski A. Arai Y. Nomura H. Baird M. Dalrymple I. (2017). Pharmacokinetics, safety, tolerability and immunogenicity of FKB327, a new biosimilar medicine of adalimumab/Humira, in healthy subjects. Br. J. Clin. Pharmacol. 83, 1405–1415. 10.1111/bcp.13245 28133772 PMC5465341

[B13] Scheinfeld N. (2005). Adalimumab: a review of side effects. Expert Opin. Drug Saf. 4, 637–641. 10.1517/14740338.4.4.637 16011443

[B14] Shin D. Lee Y. Kim H. Körnicke T. Fuhr R. (2017). A randomized phase I comparative pharmacokinetic study comparing SB5 with reference adalimumab in healthy volunteers. J. Clin. Pharm. Ther. 42, 672–678. 10.1111/jcpt.12583 28675520

[B15] US Food and Drug Administration (2015). Scientific considerations in demonstrating biosimilarity to a reference product. (United States: federal Food and Drug Administiration). Available at: http://www.fda.gov/downloads/drugs/guidancecomplianceregulatoryinformation/guidances/ucm291128.pdf (Accessed April 28, 2015).

[B16] von Richter O. Lemke L. Haliduola H. Balfour A. Zehnpfennig B. Skerjanec A. (2019). Differences in immunogenicity associated with non-product related variability: insights from two pharmacokinetic studies using GP2017, an adalimumab biosimilar. Expert Opin. Biol. Ther. 19, 1057–1064. 10.1080/14712598.2019.1603959 31002537

[B17] World Health Organization (2015). Guidelines on evaluation of similar biotherapeutic products (SBPs). Available at: http://www.who.int/biologicals/areas/biological_therapeutics/BIOTHERAPEUTICS_FOR_WEB_22APRIL2010.pdf (Accessed February 4, 2015).

[B18] Wynne C. Altendorfer M. Sonderegger I. Gheyle L. Ellis-Pegler R. Buschke S. (2016). Bioequivalence, safety and immunogenicity of BI 695501, an adalimumab biosimilar candidate, compared with the reference biologic in a randomized, double-blind, active comparator phase I clinical study (VOLTAIRE®-PK) in healthy subjects. Expert Opin. Investig. Drugs 25, 1361–1370. 10.1080/13543784.2016.1255724 27813422

[B19] Zelenetz A. D. Becker P. S. (2016). The role of biosimilars. J. Natl. Compr. Canc Netw. 14, 626–629. 10.6004/jnccn.2016.0178 27226500

[B20] Zhang H. Wang F. Zhu X. Chen Y. Chen H. Li X. (2020). Antiviral activity and pharmacokinetics of the HBV capsid assembly modulator GLS4 in patients with chronic HBV infection. Clin. Infect. Dis. 10.1093/cid/ciaa961 PMC8516514 32649736

[B21] Zhang Z. Fan W. Yang G. Xu Z. Wang J. Cheng Q. (2017). Risk of *tuberculosis* in patients treated with TNF-α antagonists: a systematic review and meta-analysis of randomised controlled trials. BMJ Open 7, e012567. 10.1136/bmjopen-2016-012567 PMC5372052 28336735

